# Optimization of Passive Low Power Wireless Electromagnetic Energy Harvesters

**DOI:** 10.3390/s121013636

**Published:** 2012-08-13

**Authors:** Antwi Nimo, Dario Grgić, Leonhard M. Reindl

**Affiliations:** Department of Microsystems Engineering, Laboratory for Electrical Instrumentation, University of Freiburg, IMTEK, Georges-Köhler-Allee 106, 79110 Freiburg, Germany; E-Mails: grgic@imtek.de (D.G.); leonhard.reindl@imtek.uni-freiburg.de (L.M.R.)

**Keywords:** RF energy harvesting, wireless power transmission, coupled resonators, Schottky diode, RF to DC power converter, impedance matching, PI-matching, L-matching, rectenna

## Abstract

This work presents the optimization of antenna captured low power radio frequency (RF) to direct current (DC) power converters using Schottky diodes for powering remote wireless sensors. Linearized models using scattering parameters show that an antenna and a matched diode rectifier can be described as a form of coupled resonator with different individual resonator properties. The analytical models show that the maximum voltage gain of the coupled resonators is mainly related to the antenna, diode and load (*remote sensor*) resistances at matched conditions or resonance. The analytical models were verified with experimental results. Different passive wireless RF power harvesters offering high selectivity, broadband response and high voltage sensitivity are presented. Measured results show that with an optimal resistance of antenna and diode, it is possible to achieve high RF to DC voltage sensitivity of 0.5 V and efficiency of 20% at −30 dBm antenna input power. Additionally, a wireless harvester (*rectenna*) is built and tested for receiving range performance.

## 1. Introduction

For autonomous powering of sensor nodes in remote or inaccessible areas, wireless power transfer provides the only viable option to power them from an energy source. Due to the low power density of ambient RF at far-field from transmitters, there is a need to optimize each aspect of a wireless RF energy harvester for possible realistic applications. Today remote autonomous sensors are mostly powered by batteries, which have limited lifespan. Renewable powering has the potential to power autonomous sensors perpetually. Due to the expansion of telecommunications technology ambient electromagnetic (EM) power is among the most common sources of ambient energy. There are power transmitters/receivers scattered in practically any society, ranging from television transmission stations to cell phone transmitters and even wireless routers in our homes/offices or mobile phones. These transmitters in our environment and others which are on special dedicated frequencies produce ambient RF power (on the order of microwatts) which can be used as a source for powering remote microwatt budget sensors through wireless energy harvesting. This work presents different matching techniques based on different application requirements using Schottky diode-based RF to DC power converting circuits for wireless remote EM energy harvesting around 434 MHz and 13.6 MHz. Generalized analytical models and limitations of the matched RF to DC power converters are discussed. A wireless RF energy harvester consisting of an antenna and a matched diode rectifier is then realized and its performance tested. Passive wireless energy harvesting also finds applications in near field communications (NFC) [1], RFID tags [2–5], implantable electronics [6,7], and environmental monitoring [8], among others.

### 1.1. State of the Art

Hertz was the first to demonstrate the propagation of EM waves in free space and to demonstrate other properties of EM waves such as reflection using parabolic reflectors [9]. Wireless power transmission was then investigated and demonstrated for possible wireless remote powering by Tesla. Electromagnetic power beaming for far field wireless power transfer using collimated EM waves was proposed in the 1950s [9]. Recent advances in ultralow power sensors means ambient omni-directional EM power can be used as a source for powering remote sensors without the need to collimate the EM power through the wireless space. Mickle [10] and McSpadden [11] have presented earlier work on wireless energy harvesting systems using Schottky diodes and rectennas where the usability of ambient RF power into DC power was shown. Sample [12] presented a wireless harvester which can harvest EM power from TV and radio base stations transmitting 960 kW of effective radiated power; 60 μW was harvested at a range of about 4 km. Umeda [13] and Le [14] have presented more integrated wireless energy harvesters based on CMOS RF to DC rectifying circuits. CMOS-based rectifying power converters provide full compatibility with standard CMOS technologies and have advantages in batch processes for mass production. The drawback of CMOS-based diode connected transistors is the need to bias the gate of the transistors for the rectifying circuits to effectively function. This gate bias is provided externally, which makes the system not passive. Without the injection of external charges or a biasing of the transistor gate, the circuit has low efficiency, especially when the amplitude of the input voltage is low [15]. Shameli [2] presented a passive CMOS RF to DC power converter with a voltage sensitivity of 1 V at −14.1 dBm input, but the circuit efficiency was only 5 %. Zbitou [16] presented an RF to DC converter based on Schottky diodes and achieved 68 % efficiency at 20 dBm RF input power. Ungan [17,18] presented antennas and high quality factor RF to DC power converters at 24 MHz and 300 MHz for RF wireless energy harvesting at 30 dBm input power. The power converter used high quality factor resonators for impedance matching the EM source and the diodes and achieved high open circuit voltage sensitivity of 1 V/μW. Boquete [19] presented a risk assessment system for calculating insurance premiums by monitoring mobile phone usage while driving. This was done by harvesting EM power from detected mobile phone usage during driving for risk assessment. Heikkinen [20] presented rectennas on different substrates at 2.4 GHz using transmisson lines to match the antennas output resistance (*at resonance*) to the rectifying diodes. Akkermans [21] presented a rectenna design by complex conjugating impedance provided by a microstrip structure to a diode so that resonance may be achieved for a working frequency. This design approach may need sophisticated tools to realize and the dominant resonance frequency of the rectenna can be unpredictable in practice. Hagerty [22] presented rectenna arrays for broadband ambient EM harvesting and characterized the harvesters from 2 GHz to 18 GHz; rectennas combine impedance matching the RF rectifying circuit and the antenna into one compact device, but an array of rectennas may increase the overall size of an EM harvester. Herb [23] and Vullers [24] have provided a comprehensive state of the art for micro energy harvesting and have explored the various techniques used for harvesting ambient renewable energy.

## 2. RF to DC Power Converter

### 2.1. Diode Rectifier

**Figure 1.**
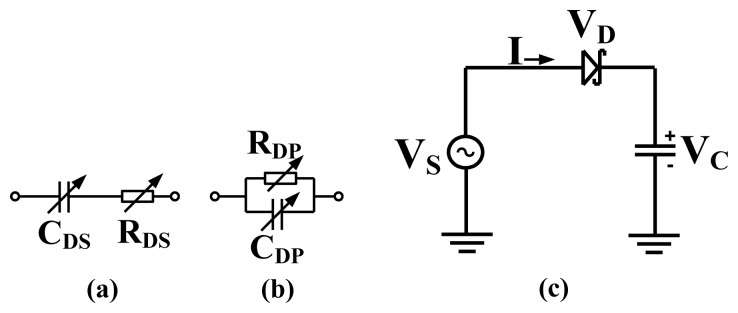
(**a**) Diode series equivalent model, (**b**) Diode parallel equivalent model, (**c**) Simple diode detector.

A junction diode equivalent circuit and simple Schottky diode rectifier are shown in Figure 1. *R_DS_* is the diode resultant series resistance, *C_DS_* is the diode resultant series capacitance, *R_DP_* is the diode resultant parallel resistance, *C_DP_* is the diode resultant parallel capacitance, *Vs* is the sinusoidal source voltage and *Vc* is the voltage across the capacitor.

The diode capacitive impedance is mainly due to the junction capacitances provided by the metal, its passivation and the semiconductor forming the diode. AC power incident on a forward biased diode input is converted to DC power at the output. The current-voltage behavior of a single metal/semiconductor diode is described by the Richardson equation [25] as in Equation (1):

(1) $$I=I_{S}\left(e^{\left(\overset{qV_{D}}{\;}/\underset{n\text{KT}}{\;}\right)}-1\right)$$

where *I* is the current through the diode, *I_S_* is the saturation current, *q* is the charge of an electron, *V_D_* is the voltage across the diode, *T* is the temperature in degrees Kelvin and *K* is Boltzmann constant. The voltage equation around the loop can be derived from Figure 1(c) and is given in Equation (2):

(2) $$V_{D}=V_{S}-V_{C}$$

Since the same current flows through the diode and the capacitor, one can find the average current through the circuit by integrating Equation (1) over a time period. By substituting Equation (2) into Equation (1), *V_C_* can be expressed in terms of *V_S_* by averaging the diode current to zero. This is given in Equation (3) [26]:

(3) $$V_{C}=\frac{\text{KT}}{q}\ln\left[\vartheta_{0}\left(\frac{qV_{S}}{\text{KT}}\right)\right],$$

where *ϑ*_0_ is the series expansion of the sinusoidal source voltage. Equation (3) can further be simplified for very small amplitude *V_S_* as Equation (4):

(4) $$V_{C}\approx \frac{qV_{S}^{2}}{4\text{KT}}$$

Equation (4) shows that for a small voltage source, the circuit output voltage is proportional to the square of the input sinusoidal voltage; hence it's so-called square law operation. Extensions of this model for voltage multipliers and other input signals are presented in [27] and [28]. Equation (4) further confirms that for low input voltage (power ≤ 10 dBm), an impedance matching network between the source and the diode is necessary to improve the detected output voltage and efficiency.

### 2.2. Impedance Matching

The maximum power transfer theorem states that the highest power is transferred to the load when the source resistance is the same as the load resistance. For systems with both resistive and reactive impedances from source and load, the source and the load impedance should be adjusted in a way that they are the complex conjugate of each other through impedance matching. For the purposes of this work, a 50 Ω resistive source is chosen as reference for load impedance matching. The antenna which captures the ambient RF signals is tuned to provide this source resistance at resonance for the rectifying circuit in a complete EM wireless remote harvester. The load is the resistance of the Schottky diodes and the actual connected resistance (*remote sensor*). The specific type of matching network which can be used for complex conjugation depends on the nature of load or source impedance, the desired RF to DC converter functionality and other factors like circuit size, cost, *etc.* The response of a matched RF to DC power converter depends on the matching network used as well as the source or load component quality factors and impedances.

### 2.3. Diode Impedance

**Figure 2.**
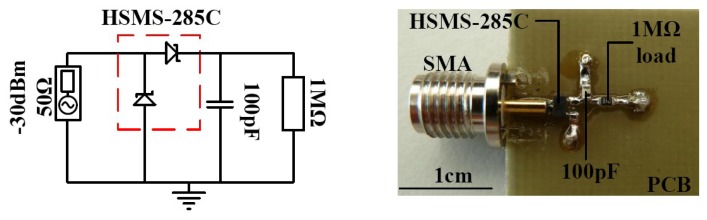
(**left**) Reference circuit layout for measuring diodes input impedance, (**right**) measuring printed circuit board (PCB) for diodes input impedance on 1 mm FR4 substrate.

Schottky diodes HSMS-285C and HSMS-286C from Avago [29,30] are used to build the RF to DC power converters. The HSMS-285× or 286× series diodes can be operated as zero biased with relatively low forward junction potential. This allows for the realization of completely passive RF to DC power converters for wireless energy harvesting. The HSMS-285C or 286C is a pair of series connected Schottky diodes in a SOT-323 package. The impedance of the HSMS-285C and HSMS-286C diodes was first measured so it can be matched to the resistance (50 Ω) of the antenna source. This is done by connecting the input of the diodes to a network analyzer and measuring the scattering parameters. These scattering parameters are then converted to the corresponding impedances. The input impedance of a diode depends mainly on the resistive and capacitive impedance provided by the junction of the diode and its connected load. For a couple of diodes arranged in a package such as the HSMS-285C or 286C, the input impedance is the vector sum of the impedances provided by each diode in the package arrangement, the extra impedance associated with the packaging and the connected load. The diode measuring board is as shown in Figure 2. The diodes were measured at room temperature for an input power of 30 dBm at a diode connected load of 1 MΩ with a 100 pF filter capacitor. For the sake of this work, the input impedance of the diodes will always be referred to at these connected load conditions.

**Figure 3.**
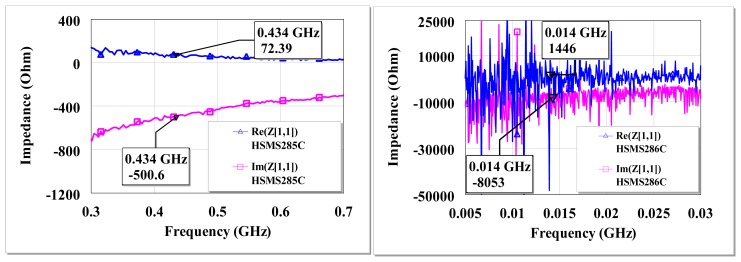
Measured input impedance (Δ resistive, □ capacitive) of HSMS-285C (**left**) and HSMS-286C (**right**) diodes at −30 dBm input with 1 MΩ load and 100 pF filter.

The board is fabricated such that components are soldered directly one into another to prevent additional impedances introduced by copper route. The PCB backside had the ground layer. An example of measured input impedance for HSMS-285C and HSMS-286C is shown in Figure 3.

The diodes quality factor is given by *X_DS_R_DS_*^−1^, where *X_DS_* is the resultant series capacitive impedance of the diodes. At an input power of −30 dBm, the measured input impedance of the HSMS-285C diodes is 72–j501 Ω at 434 MHz and 587–j1239 Ω at 13.6 MHz. For HSMS-286C diodes, it is 10–j503 Ω at 434 MHz and ∼1.5–j8.1 kΩ at 13.6 MHz for −30 dBm input. The measured impedance of the HSMS-286C diodes at low frequencies (< 60 MHz) shows pronounced fluctuations. The low-frequency excess flicker noise and the shot noise observed in the HSMS-286C have been studied by several authors [31–33]. The pronounced presence of trap states in the depletion region of the semiconductor, mobility fluctuations in carriers, edge effects among other reasons is reported to cause deviations from the ideal Schottky diode behavior and hence generation-recombination noise for some diodes such as the HSMS-286C [34]. When a diode rectifier is matched at a reference operating condition, the matching network may function less effectively at other input power levels, connected load and other operating frequencies. This is due to possible changes in the diode input impedance. Throughout this work the imperfections of the matching circuit at other operating conditions away from the matched reference conditions are accepted without changes to the matching network.

### 2.4. Voltage Doubler

**Figure 4.**
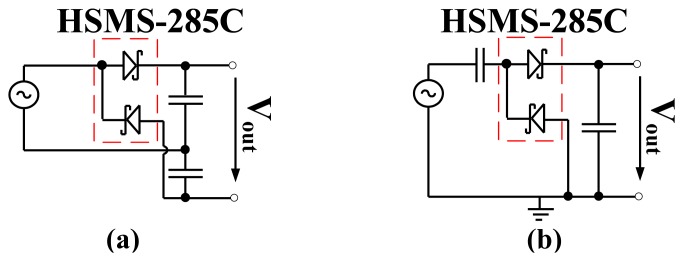
Circuit diagram of voltage doubler, (**a**) Delon doubler and (**b**) Greinacher doubler.

The Delon voltage doubler and Greinacher doubler are both used to realize the RF to DC power converters presented in this work. The Delon voltage doubler and Greinacher doubler are shown in Figure 4. The diodes output voltage (*V_out_*) is doubled what is detected by a simple detector circuit shown in Figure 1. Both doublers produce the same output performance, the only difference is that the Delon doubler has an instantaneous input ground which is not shared with the output.

### 2.5. Matching Techniques for Antenna Source and RF to DC Power Converter

#### 2.5.1. L-match RF to DC Power Converter

An L-match network converts a source series impedance to its equivalent load parallel impedance or *vice-versa* and tunes out by subtracting or adding any surplus reactance from the load or source with the counter impedance. Series impedance is converted to its parallel equivalent impedance using Equations (5–7):

(5) $$Q_{S}=\frac{X_{S}}{R_{S}}$$

(6) $$Q_{P}=\frac{R_{p}}{X_{P}}$$

where *Xs* is the total series reactive impedance, *Rs* is the total series resistance, *R_P_* is the total parallel resistance, *Xp* is the total parallel reactive impedance, *Q_S_* and *Qp* are the series and parallel quality factors respectively:

(7) $$R_{S}+jX_{S}=\frac{R_{P}\times jX_{P}}{R_{P}+jX_{P}}$$

Equation (7) is the equation of a series sum of impedances and a parallel sum of impedances. It is interesting to note that *Q_S_* and *Q_P_* from an L-matched network may be different from the individual component quality factors as a result of the inherent resistive and reactive impedances in that component. By virtue of Equation (7), *Q_S_* and *Q_P_* must be equal in an L-matched network. Using Equations (5,6) and (7), the ratio of the parallel resistance (*or reactance*) to the series resistance (*or reactance*) can be derived in terms of the quality factors *Q_P_* or *Q_S_* [35]. Since at match conditions, only the resistive impedances dissipate power, the loaded quality factor *Q*, of the L-matched network can be expressed as in Equation (8):

(8) $$R_{P}=(Q^{2}+1)R_{S}$$

Using Equations (5,6) and (8), series impedance can be converted to its parallel equivalent for a fixed frequency and power level. As an example; a series impedance 72–j501 Ω (HSMS-285C at 434 MHz for −30 dBm input power) is easily converted to −*j*510(3519)/(−*j*510 + 3519) Ω as its parallel equivalent with a component quality factor of 6.96. The source resistance is taken as part of the parallel matching network in an L-match circuit if the source series equivalent resistance is greater than the load series equivalent resistance. On the other hand, the load resistance is taken as part of the parallel matching network if the load series equivalent resistance is greater than the source series equivalent resistance. For the purpose of this work, inductors were only used for series impedance matching and capacitors as shunts. This prevents power seeping through any shunt inductor used for impedance matching due the short circuit provided by a shunt inductor to ground and resulting in less output efficiency. Resistors were not used for impedance matching.

#### 2.5.2. L-match RF to DC Converter Generalized Analytical Model

**Figure 5.**
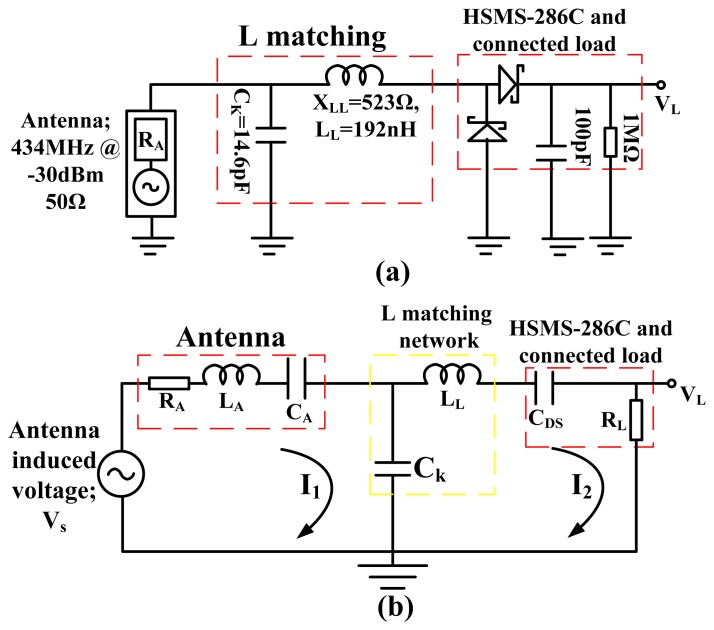
(**a**) L-match RF to DC harvester using the HSMS-286C diodes at 434 MHz for −30 dBm input. (**b**) Small signal impedance model of a generalized L-matched RF to DC power converter as capacitive coupled series RLC resonator with different resonator elements.

The classical matching technique using Equations (5,6) and (8) is first used to L-match the 50 Ω resistance of the antenna to the resistance of the HSMS-286C diodes (and load) at 434 MHz for −30 dBm input and then the generalized model is discussed. The antenna source resistance was L-matched to the resistance of the diodes (and load). The 50 Ω resistance of the antenna is taken as the parallel matching component and the diodes 10 Ω resistance is the series matching component. The loaded *Q* is found as 2 between the 50 Ω antenna source resistance and the 10 Ω diode series resistance using Equation (8). From this loaded *Q*, a shunt capacitive impedance of 25 Ω (14.6 pF at 434 MHz) using Equation (6) and a series inductive impedance of 20 Ω (7.3 nH at 434 MHz) using Equation (5) will match the 50 Ω source to the 10 Ω HSMS-286C diodes (and load) series resistance at −30 dBm input. Since the HSMS-286C diodes inherently provides 503 Ω series capacitive impedance at −30 dBm, a resultant series inductive impedance of 523 Ω (192 nH at 434 MHz) is needed to tune the 50 Ω resistive source to the complete HSMS-286C diodes impedance at 434 MHz for −30 dBm input. The L-matched HSMS-286C diodes rectifier is as shown in Figure 5(a).

*C_K_* is the tuning capacitance, *L_L_* is the tuning inductance, *X_LL_* is the tuning inductive impedance, *C_DS_* is the diodes series capacitance, *X_DS_* is the diodes series capacitive impedance, *V_S_* is the antenna captured ambient EM voltage, *R_A_* is the resistance of antenna, *L_A_* is the inductance of antenna, *C_A_* is the capacitance of antenna, *R_L_* is the resultant series resistance from the diodes and the connected load resistance, *V_L_* is the resistive load voltage. From Figure 5(a) the power dissipated in the resistance of the diodes (and connected load); P_L_ is given by Equation (9), where R_L_ is the series resistance of the diodes and load:

(9) $$P_{L}=\frac{V_{L}^{2}}{R_{L}}$$

The source power; P_S_ is given by Equation (10), where V_S_* is the root mean squared (RMS) antenna captured source voltage. Half of the source power is transferred to the resistance of the diodes (and connected load) at match conditions as described by the maximum power transfer theorem:

(10) $$\begin{array}{cc} P_{S}=\frac{V_{S}^{2}}{R_{A}} & \text{or}\;P_{S}=\frac{{V_{S*}}^{2}}{2R_{A}} \end{array}$$

Equating *P_L_* and half RMS *P_S_* gives a condition of maximum voltage gain for the matched RF to DC power converter shown in Figure 5(a):

(11) $$\frac{V_{L}}{V_{S*}}=\frac{1}{2}\sqrt{\frac{R_{L}}{R_{A}}}$$

From Equation (8), substituting the series and parallel resistance ratio into Equation (11) the voltage gain can be expressed in terms of the loaded quality factor as in Equations (12) and (13), where *Q* is the loaded quality factor of the RF to DC power converter:

(12) $$\frac{V_{L}}{V_{S*}}=\frac{1}{2}\sqrt{\frac{1}{1+Q^{2}}}$$

Equation (12) is the voltage gain in-terms of the loaded *Q* if the resistance of the diodes (and connected load) is part of the series matching network and the resistance of the antenna source is part of the parallel matching network as in Figure 5(a). If the resistance of the diodes is part of the parallel matching network, then Equation (13) may be written as the voltage gain in-terms of the loaded *Q* in an L-matched circuit:

(13) $$\frac{V_{L}}{V_{S*}}=\frac{1}{2}\sqrt{1+Q^{2}}$$

Equations (12) and (13) shows that the maximum voltage gain is directly related to the relative differences between the diodes (and connected load) resistance and source resistance at matched conditions or the circuit loaded quality factor. It is interesting to note that the circuit shown in Figure 5(a) has a loaded Q of 2, but an HSMS-286C unloaded quality factor of 50 (at 434 MHz for −30 dBm).

Figure 5(a) is generally modeled as capacitive coupling of two series RLC resonators with a voltage source. This linearized model can be made at any defined frequency and power level. The model however neglects the metal/semiconductor physics of the diode's junction potentials which results in a Schottky barrier. The first series RLC resonator is modeled as impedance from the antenna with or without some passive matching components. The voltage source *V_S_*, is the antenna captured electromagnetic voltage. The second series RLC resonator is the impedance from the diodes (at a defined condition), connected resistance and some passive matching components. Ck is modeled as the coupling element between the two series RLC resonators. Figure 5(b) gives a more general look at the special scenario shown in Figure 5(a). The voltage equations in the two loops are given by Equations (14,15) according to Kirchhoff's voltage loop laws, where *ω* is the angular frequency and *I_1_*, *I_2_* are the currents in the first loop and second loop, respectively:

(14) $$V_{S}=I_{1}\left[R_{A}+j\omega L_{A}-\frac{j}{\omega C_{A}}-\frac{j}{\omega C_{K}}\right]+\frac{jI_{2}}{\omega C_{K}}$$

(15) $$0=\frac{jI_{1}}{\omega C_{K}}+I_{2}\left[R_{L}+j\omega L_{L}-\frac{j}{\omega C_{DS}}-\frac{j}{\omega C_{K}}\right]$$

Using Cramers rule, *I_2_* can be expressed as:

(16) $$I_{2}=\frac{\frac{-jV_{S}}{\omega C_{K}}}{\left[R_{A}+j\omega L_{A}-\frac{j}{\omega C_{A}}-\frac{j}{\omega C_{K}}\right]\left[R_{L}+j\omega L_{L}-\frac{j}{\omega C_{DS}}-\frac{j}{\omega C_{K}}\right]+\frac{1}{\omega^{2}C_{K}^{2}}}.$$

The voltage across *R_L_* is *V_L_*; given by *I_2_R_L_*:

(17) $$V_{L}=\frac{\frac{-jV_{S}}{\omega C_{K}}R_{L}}{\left[R_{A}+j\omega L_{A}-\frac{j}{\omega C_{A}}-\frac{j}{\omega C_{K}}\right]\left[R_{L}+j\omega L_{L}-\frac{j}{\omega C_{DS}}-\frac{j}{\omega C_{K}}\right]+\frac{1}{\omega^{2}C_{K}^{2}}}$$

The voltage gain of the coupled resonator can be expressed as in Equation (18):

(18) $$\frac{V_{L}}{V_{S}}=\frac{\frac{-jR_{L}}{\omega C_{K}}}{\left[R_{A}+j\omega L_{A}-\frac{j}{\omega C_{A}}-\frac{j}{\omega C_{K}}\right]\left[R_{L}+j\omega L_{L}-\frac{j}{\omega C_{DS}}-\frac{j}{\omega C_{K}}\right]+\frac{1}{\omega^{2}C_{K}^{2}}}$$

At resonance, there is no resultant reactance in the RLC resonators or the capacitive and inductive impedances become equal; hence Equation (19) can be written:

(19) $$\omega L_{A}-\frac{1}{\omega }\left\{\frac{1}{C_{A}}+\frac{1}{C_{K}}\right\}=0\;\text{and}\;\omega L_{L}-\frac{1}{\omega }\left\{\frac{1}{C_{DS}}+\frac{1}{C_{K}}\right\}=0$$

Equations in Equation (19) can be used to find the resonant frequencies of the series coupled resonator. The voltage gain of the coupled resonator at resonance can then be expressed as in Equation (20):

(20) $$\frac{V_{L}}{V_{S}}=V_{\text{gain}}=\frac{\frac{-jR_{L}}{\omega C_{K}}}{R_{A}R_{L}+\frac{1}{\omega^{2}C_{K}^{2}}}$$

where *V*_gain_ is the voltage gain. *V_gain_* at resonance is a function of the resistance of the source and load and the coupling element. The maximum of Equation (20) is obtained when:

(21) $$\frac{dV_{\text{gain}}}{dC_{K}}=0.$$

This gives the results as in Equation (22):

(22) $$\frac{dV_{\text{gain}}}{dC_{K}}=\frac{j2R_{L}}{\omega^{3}C_{K}^{4}}-j\left\{R_{A}R_{L}+\frac{1}{\omega^{2}C_{K}^{2}}\right\}\frac{R}{\omega C_{K}^{2}}=0\;\text{or}\;R_{A}R_{L}^{2}=\frac{R_{L}}{\omega^{2}C_{K}^{2}}$$

Equation (22) can be simplified to find *C_K(max)_*:

(23) $$C_{K(\max)}=\pm \frac{1}{\omega }\sqrt{\frac{1}{R_{A}R_{L}}}$$

where *C_Kmax_* is the value of the coupling element where maximum power transfer from the first resonator to the second resonator occurs. Using Equations (19) and (23) the unknown optimal matching impedances can be found from the known impedances just like the classical L-matched procedure using Equations (5,6) and (8). By substituting *C_K(max)_* into Equation (20) and taking the magnitude of *V_gain_*, gives the maximum voltage gain of the coupled series resonator at resonance:

(24) $$\left|\frac{V_{L}}{V_{S}}\right|=\frac{1}{2}\sqrt{\frac{R_{L}}{R_{A}}}\;\text{or simply}\;\frac{V_{L}}{V_{S*}}=\frac{1}{2}\sqrt{\frac{R_{L}}{R_{A}}}$$

For wireless harvesters consisting of an antenna and a diode rectifying circuit, the diode resistive impedance at any condition is dependent on the diode realized parameters, signal frequency, connected load and the input power level. The source impedance is determined by the impedance of the antenna. For maximum efficiency, the ratio of the source resistance to the load resistance must tend to zero at matched conditions. The efficiency *η* of the circuit is given by Equation (25):

(25) $$\eta =\frac{P_{L}}{P_{S}};\eta \to 1\;\text{when}\;\frac{R_{A}}{R_{L}}\to 0$$

#### 2.5.3. L-Match RF to DC Converter Experimental Results and Discussion

**Figure 6.**
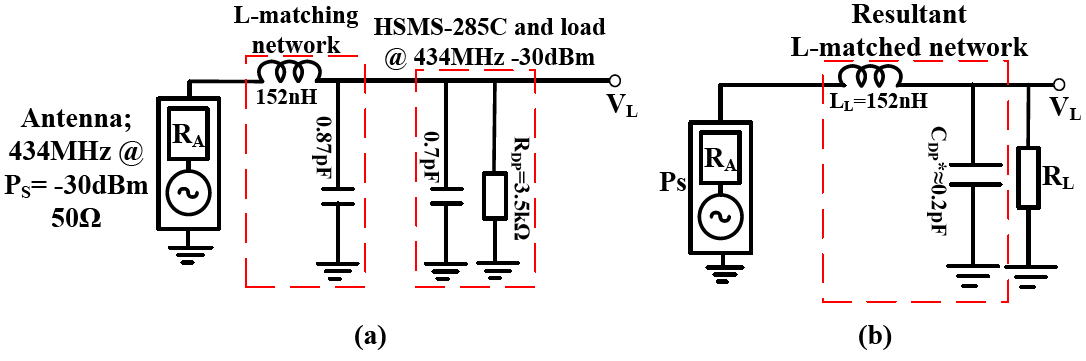

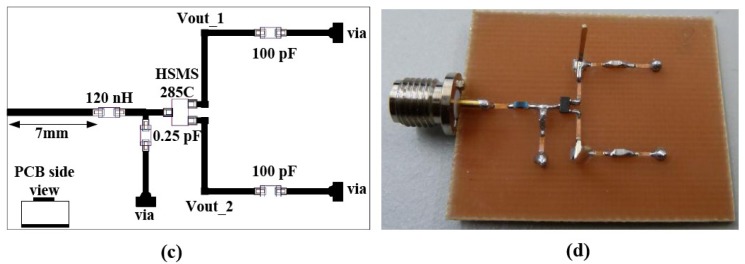
(**a**) L-matched impedance circuit for matching the HSMS-285C diodes at 434 MHz for −30 dBm input. (**b**) Resultant network, (**c**) PCB layout of the L-matched Delon doubler with adjusted values on FR4 substrate (**d**) Fabricated PCB of the L-network matched Delon voltage doubler.

The presented circuit was L-matched between the 50 Ω resistance of the antenna source and the resistance of the HSMS-285C diodes (and load) at 434 MHz for −30 dBm input as shown in Figure 6. Since the series equivalent resistance of the HSMS-285C diodes and load (72 Ω) is greater than the 50 Ω series resistive antenna source, the diode is taken as parallel matching network with a parallel equivalent impedance of −*j*510(3519)/(−*j*510 + 3519) Ω. The analysis follows the same procedure as in Section 2.5.2 after this step. Figure 6(b) shows the resultant L-matched RF to DC power converter. C_DP_* is the resultant shunt matching capacitance.

Figure 6(a,b) assume perfect characteristic impedance between the various components in the matched circuit. When a copper route is introduced between components and on a material substrate, it must be accounted for in the total impedance as seen by the source or load. This PCB impedance compensation is carried out in Advance Design Systems (ADS) from Agilent [36]. ADS has extensive models for microstrip substrates to account for its impedances. The optimized layout using ADS microstrip models and its compensated values in the passive tuning components for a Delon doubler is shown in Figure 6(c).

**Figure 7.**
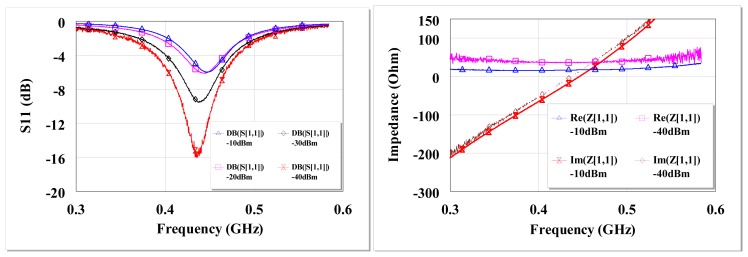
Measured open circuit S_11_ of the L-matched Delon circuit at different input power levels from a 50 Ω source (**left**), measured open circuit input impedance at −10 dBm and −40 dBm of the L-matched circuit (**right**).

The circuit reflection coefficient (S_11_) and input impedance at open circuit are shown in Figure 7. There is high return loss and resonance around 434 MHz. The circuit input impedance at open circuit conditions is ∼38 Ω at resonance for −40 dBm and ∼17 Ω at −10 dBm input.

**Figure 8.**
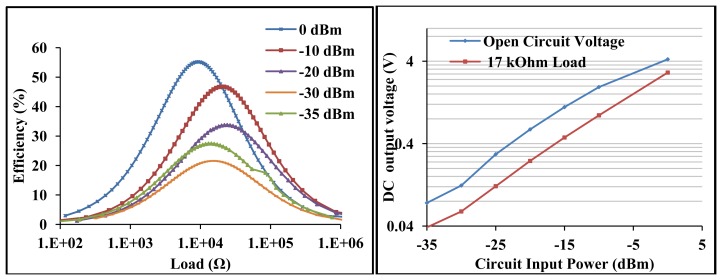
Measured L-matched circuit efficiency *versus* resistive load at various input power levels at 434 MHz (**left**), measured open circuit voltage and at 17 kΩ load *versus* input power at 434 MHz (**right**).

The measured L-matched circuit efficiency and voltage sensitivity is shown in Figure 8. The maximum measured L-matched efficiency at −30 dBm is 22% at ∼20 kΩ load and an open circuit voltage of 124 mV. At −10 dBm, the maximum efficiency and open circuit voltage is 47% and 2 V respectively. At the optimal load of ∼20 kΩ, the detected voltage is 58 mV and 1 V at −30 dBm and −10 dBm respectively.

The open circuit voltage gain is 25 at −30 dBm and 40 at −10 dBm. The maximum measured efficiency at −35 dBm is 27%. This is higher than that of −30 dBm due to the better matched circuit impedance at −35 dBm (35 Ω) than at −30 dBm (27 Ω) input. The L-matched RF to DC power converter has a loaded *Q*, sensitivity and efficiency determined mainly by the diodes resistance, diodes junction potential, connected resistance and antenna source resistance at matched conditions.

#### 2.5.4. PI-match RF to DC Power Converter

A highly selective or small frequency bandwidth RF power converter is realized with a PI-network in-between the source impedance from the antenna and the diode rectifier. A PI-network is a ‘back to back’ L-network that are both configured to match the load and source impedance to an invisible resistance located at the junction between the two L-networks [37]. The quality factor of the L-network with the parallel resistance is given by Equation (26):

(26) $${Q_{P}}^{*}=\sqrt{\frac{R_{P}}{R^{*}}-1},$$

where *R_P_* is the parallel resistance, R* is a virtual resistance and Q_P_* is the quality factor of the L-network with the parallel resistance. The quality factor of the L-network with the series resistance is given by Equation (27):

(27) $${Q_{S}}^{*}=\sqrt{\frac{R_{S}}{R^{*}}-1},$$

where Q_S_* is the quality factor of the L-network with the series resistance. The unloaded quality factor; Q_S_* or Q_P_* is set higher than what is normally achieved with a single L-network [37] to realize the small frequency bandwidth circuit. The resistance of the load is assigned the parallel network in a PI-matched circuit if its series equivalent resistance is higher than the source series equivalent resistance; the opposite is true if the source is higher than the load. Equation (26) and Equation (27) are synonymous to Equation (8), except the lowest resistive impedance in Equation (8) is substituted with the virtual resistance which is dependent on the newly desired circuit selectivity. From Equations (26) and (27) the loaded quality factor of the PI-matched circuit can be written as Equation (34) in terms of Q_S_* and Q_P_*:

(28) $$Q^{2}=\left[\left(\frac{Q_{P}^{*2}+1}{Q_{S}^{*2}+1}\right)-1\right],$$

where *Q* is the loaded quality factor of the PI-network. Q_S_* or Q_P_* are the unloaded quality factors of the PI-matched network. The larger value among the unloaded quality factors result in small frequency bandwidth response which is desired when matching a source and load impedance with a PI-network. Some authors approximate the highest value of Q_S_* or Q_P_* or their algebraic sum as the loaded quality factor of the PI-network as in [35] and [37], but Equation (28) gives the exact loaded *Q* of the PI-matched circuit in terms Q_S_* and Q_P_*. This allows for the correct estimation of the maximum voltage gain from the loaded quality factor.

#### 2.5.5. Selectivity RF to DC Converter Generalized Analytical Model

**Figure 9.**
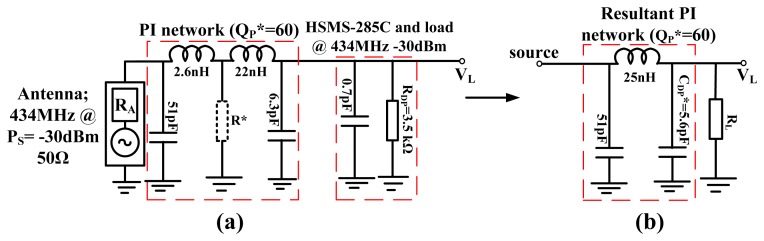
Impedance diagram of PI-matched RF power converter; (**a**) Impedance diagram of 50 Ω source and the HSMS-285C diodes at 434 MHz, (**b**) Resultant PI matched network between the antenna source and load resistance.

An example of a PI-matched RF to DC converter using the HSMS-285C diodes operating at 434 MHz for −30 dBm input is presented first and then the generalized model is discussed. The circuit is matched for Q_P_* of 60 between the antenna and the resistance of the diodes as shown in Figure 9.

**Figure 10.**
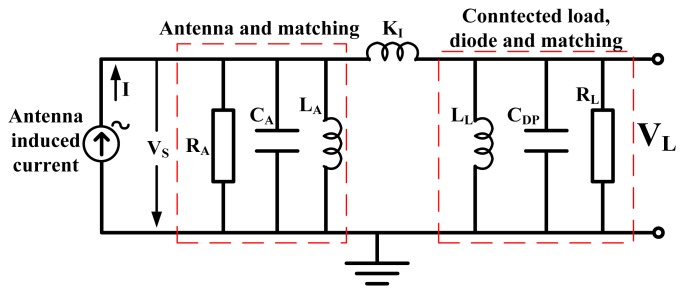
Inductive coupled parallel RLC small signal model of a generalized PI-matched antenna and diode rectifier.

Figure 9(b) can also be modeled as an inductive coupling of two parallel RC circuits. A more general look at such a circuit is shown in Figure 10, as an inductive coupling of two parallel RLC resonators with a current source.

The first parallel RLC resonator is modeled as impedance from the antenna and some passive matching components. The second parallel RLC resonator is modeled as impedance from the linearized diodes, its connected load and some passive matching components. *I* is the antenna induced current, *V_S_* this time is the voltage across the parallel *R_A_* and *K_1_* is the coupling element between the two parallel resonators. Using Kirchoff's current laws, the node equations can be expressed as Equations (29) and (30):

(29) $$I=V_{S}\left[\frac{1}{R_{A}}+j\omega C_{A}-\frac{j}{\omega L_{A}}-\frac{j}{\omega K_{1}}\right]+\frac{jV_{L}}{\omega K_{1}}$$

(30) $$0=\frac{jV_{S}}{\omega K_{1}}+V_{L}\left[\frac{1}{R_{L}}+j\omega C_{DP}-\frac{j}{\omega L_{L}}-\frac{j}{\omega K_{1}}\right]$$

Load voltage (*V_L_*) and the source voltage (*V_S_*) at resonance are given by the equations in Equation (31). The resonance frequencies are given by Equation (32):

(31) $$\begin{array}{ccc} V_{L}=\frac{\frac{-jI}{\omega K_{1}}}{\left[\frac{1}{R_{A}R_{L}}+\frac{1}{\omega^{2}K_{1}^{2}}\right]} & \;\text{and}\; & V_{S}= \end{array}\frac{\frac{I}{R_{L}}}{\left[\frac{1}{R_{A}R_{L}}+\frac{1}{\omega^{2}K_{1}^{2}}\right]}$$

(32) $$\omega C_{A}-\frac{1}{\omega }\left\{\frac{1}{L_{A}}+\frac{1}{K_{1}}\right\}=0\;\text{and}\;\omega C_{DP}-\frac{1}{\omega }\left\{\frac{1}{L_{L}}+\frac{1}{K_{1}}\right\}=0$$

From *V_L_* and *Vs* in Equation (31), the voltage gain at resonance can be expressed as:

(33) $$\frac{V_{L}}{V_{S}}=\frac{R_{L}}{j\omega K_{1}}$$

The maximum of Equation (33) is obtained when:

(34) $$\begin{array}{ccc} j\omega K_{1}\to 0 & or & R_{L}\to \infty \end{array}$$

Since *jωK_1_* is restricted by the conditions in Equation (32) to attain resonance, one cannot manipulate *jωK_1_* alone without changing the resonance conditions. What can drive the voltage gain is if *R_L_* is very large at resonance conditions. If the input impedance (*V_S_/I*) of the coupled resonator is maximum at resonance, conditions in Equation (35) hold:

(35) $$\left(\frac{V_{S}}{I}\right)\to \;\text{maximum when}\;\frac{R_{L}}{\omega^{2}K_{1}^{2}}\to 0$$

Equation (36) may be assumed when $\frac{R_{L}}{\omega^{2}K_{1}^{2}}\to 0$ :

(36) $$\frac{V_{S}}{I}=R_{A}$$

Under these conditions and an optimal coupling coefficient *K_1max_*, the maximum voltage gain of the parallel coupled resonator can be written as in Equation (37), where *K_1max_* is given by Equation (38):

(37) $$\left|\frac{V_{L}}{V_{S}}\right|=\left|V_{\text{gain}}\right|=\left|\frac{1}{2}\sqrt{\frac{R_{L}}{R_{A}}}\right|$$

(38) $$K_{1(\max)}=\mp \frac{1}{\omega }\sqrt{R_{A}R_{L}}$$

The analysis of Section 2.5.2 and parallel coupled RLC resonators show that any antenna and matched rectifying diode can be described as an equivalent circuit of a coupled resonator at a defined operating point. This general model can be applied to optimize other harvesters with complex output impedance such as piezo-harvesters or vibration harvesters for maximum transfer of power or voltage to its connected load. The model can also be applied to near field magnetically coupled antennas/coils for optimization.

#### 2.5.6. Broadband RF to DC power converter

A broadband network is preferred when an RF to DC power converter is to be operated for a wide range of frequencies. A broadband converter is realized by connecting successive L-networks together in a multi-network between the antenna source and the rectifying diodes. The result is broadband or multiband RF power converter around certain frequencies. This can be deduced from the general model of a coupled resonators that by choosing certain passive components between a source and the load, it is possible to have more frequencies (*ω*) fulfilling Equation (32) and hence a result of multiple resonant frequencies or broader bandwidth at match conditions. For a two stage L-connected match, the quality factor of the L-network with the parallel resistance is given by Equation (39):

(39) $${Q_{P}}^{*}=\sqrt{\frac{R_{P}}{R^{*}}-1}$$

The quality factor of the L-network with the series resistance is given by Equation (40):

(40) $${Q_{S}}^{*}=\sqrt{\frac{R_{*}}{R_{S}}-1}$$

From Equations (39) and (40) the loaded quality factor of the two stage L-connected broadband network may be written as Equation (41) in terms of the unloaded quality factors; Q_S_* and Q_P_*:

(41) $$Q^{2}=\{(Q_{P}^{*2}+1)(Q_{S}^{*2}+1)\}-1$$

R* in this case may be chosen if it is larger than *R_S_* and lower than the *R_P_*. The highest possible bandwidth between a resistive source and resistive load is found for a virtual resistance (R*) given in Equation (42) [37]:

(42) $$R^{*}=\sqrt{R_{S}R_{P}}$$

For complex loads such as rectifying diodes or transistors, the largest achievable bandwidth prescribed by Equation (42) is limited by the load or source component quality factor, since Equation (42) does not take into account reactive impedance associated with the source or load.

#### 2.5.7. Broadband-Match RF to DC Converter Results and Discussion

**Figure 11.**
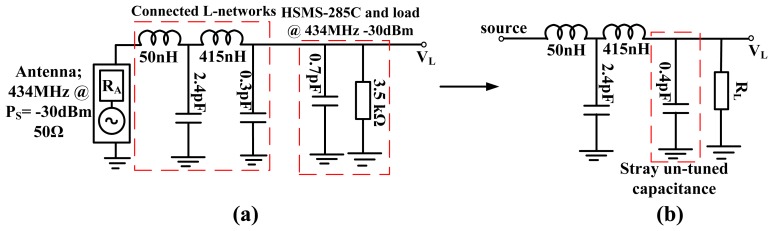
Impedance diagram of broadband RF power converter; (**a**) Broadband match around 434 MHz with loaded Q of 2.7, (**b**) Resultant impedance matching network with un-turned capacitance of 0.4 pF.

The antenna source resistance was broadband matched to the HSMS-285C diodes (and load) resistance at −30 dBm input around 434 MHz. For a desired Q_P_* and Q_S_* of 2.7 there is ∼0.4 pF inherent diode capacitance which is un-tuned using a two stage L-matching network [Figure 11(b)]. This is because the HSMS-285C diodes provides an inherent component quality factor of 6.96 at 434 MHz for −30 dBm input, hence a broadband circuit with Q_P_* lower than this inherent component quality factor of the diodes (and load) is difficult to achieve without trade-offs. However, connected L-networks with Q_P_* as high as the diode component quality factor may perform worse than a single L-matched network with similar loaded quality factor. This is due to redundant components of the connected L-networks which have inherent losses.

**Figure 12.**
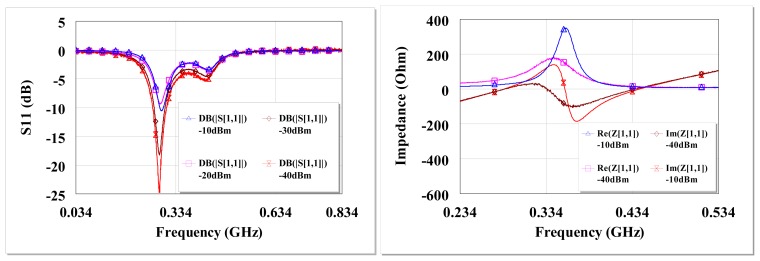
Measured open circuit S_11_ of the broadband circuit around 434 MHz at different input power levels from a 50 Ω source (**left**), measured open circuit input impedance at −10 dBm and −40 dBm of the broadband circuit (**right**).

**Figure 13.**
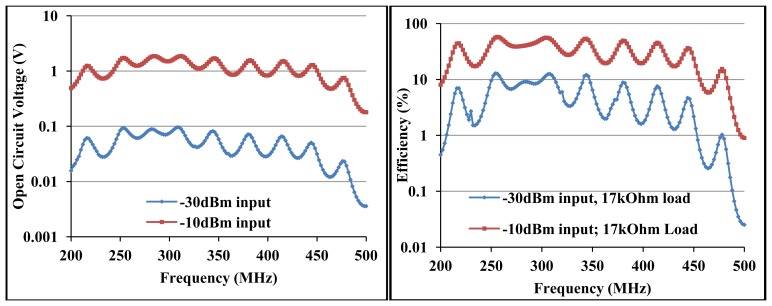
Measured open circuit voltage *versus* frequency sweep from 200 MHz to 500 MHz for −10 dBm and −30 dBm (**left**), measured efficiency at 17 kΩ load *versus* frequency sweep for −10 dBm and −30 dBm (**right**).

Therefore the broadband circuit is matched for Q_P_* of 2.7, notwithstanding the un-tuned shunt capacitance as can be seen in Figure 11(b). Figure 12 shows the circuit S_11_ at various input power levels and input impedance at open circuit conditions. From Figure 12 (left) there is ∼−5 dB return loss from 200 MHz to 500 MHz providing an operating band of ∼300 MHz. The impedance of the circuit shows resonances at ∼290 MHz and ∼450 MHz as shown in Figure 12(right). A third resonance occurs around 356 MHz at −10 dBm as the frequency of highest harvester input resistance (∼350 Ω) and where the reactive impedances approach their extremes. Figure 12 show that a wireless EM harvester can exhibit different resonance scenarios depending on the dominant instantaneous conditions. The efficiency and voltage sensitivity of the broadband matched wireless EM harvester are shown in Figure 13. The average open circuit voltage is 47 mV and 1.1 V at −30 dBm and −10 dBm, respectively, when operating from 200 MHz to 500 MHz.

The broadband circuit achieves average efficiency of 5% at 17 kΩ load for −30 dBm and 30% at 17 kΩ load for −10 dBm input power from 200 MHz to 500 MHz. Figure 13 further confirm a direct link between frequency response and the unloaded quality factors. For Q_S_* and Q_P_* of ∼2.7, the circuit response is broadband around 434 MHz.

### 2.6. High Voltage Sensitive RF to DC Converter

The current state of the art low power remote sensors would require a DC voltage supply of about 1 V and DC current of about 30 μA for operation. Therefore, the issue is not only how efficient a wireless EM harvester is in converting RF to DC power, but also what the output DC voltage and current of the EM harvester are at the RF input power level [38]. Equations (11,24) and (33) show that the maximum voltage sensitivity of a coupled resonator system or an RF to DC power converter is mostly related to the load and the source resistances at resonance. Therefore high voltage sensitive wireless EM harvester can be achieved with a diode voltage doubler with a very high input resistance relative to the antenna source without the need to cascade the diodes as in voltage multipliers. If the diodes been used for the RF to DC power conversion cannot provide high resistive impedance at the working frequency relative to the antenna source, then a DC-DC converter can be applied after the EM harvester as presented in [39] or the diodes may be cascaded by way of multipliers as presented in our earlier work [40] and by several other authors [3,5,14]. In case of multipliers, the input voltage ought to be high enough to overcome the junction potential of the several diodes in the multiplier network. If frequency is not a constraint, then a frequency sweep *versus* impedance for the diodes can be made and the frequency where the diodes exhibits high resistive impedance can be used to realize high voltage sensitive wireless RF harvester. For Schottky diodes, high resistive impedance occurs mostly at lower frequencies (see Figure 3). The measured voltage gain of a high resistive diode pair (voltage doubler) is presented in the next results.

#### 2.6.1. High Voltage Sensitive RF to DC Converter Results and Discussion

The presented result was L-matched using 50 Ω resistance of the antenna source and the resistance of the HSMS-286C diodes (and load). The HSMS-286C diodes do provide high resistive impedance at low frequencies; notwithstanding the flicker noise which causes its resistive (and reactive) impedance to fluctuate. The HSMS-286C has low forward junction potential (∼350 mV at 1 mA) per diode and series impedance of ∼1.5–j8.1 kΩ or parallel impedance of ∼−*j*8.3(46.3)/(−*j*8.3 + 46.3) kΩ at 13.6 MHz for −30 dBm input. Even though the HSMS-286C diodes unloaded component quality factor at 13.6 MHz is similar to that of the HSMS-285C diodes at 434 MHz, the elevated resistive impedance at 13.6 MHz fulfills the condition for high voltage sensitivity relative to a 50 Ω antenna source at resonance conditions.

**Figure 14.**
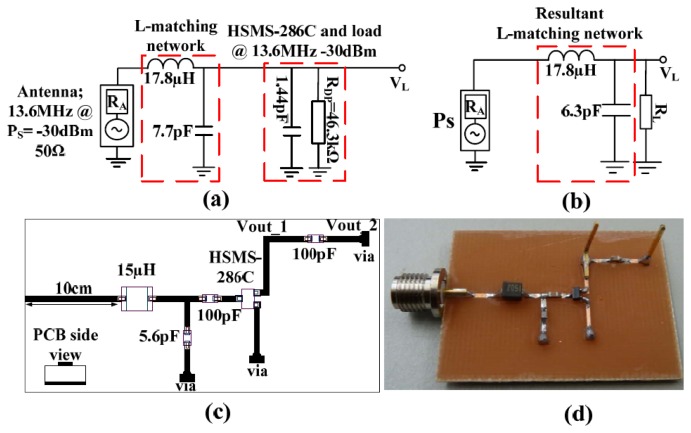
(**a**) L-matched impedance diagram for matching the HSMS-286C diodes at 13.6 MHz at -30 dBm input. (**b**) Resultant network, (**c**) PCB layout of the L-matched Greinacher doubler with adjusted values due to impedances provided by copper route on FR4 substrate with thickness of 1 mm. (**d**) Fabricated PCB of the L-matched RF to DC power converter.

The high voltage sensitive EM harvester operating at 13.6 MHz is as shown in Figure 14. On the realized PCB is a Greinacher doubler. An inductance of 15 μH and a shunt capacitance of 5.6 pF were the adjusted values after the microstrip contributions.

**Figure 15.**
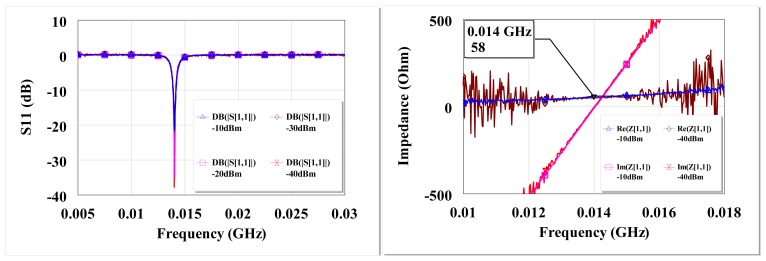
Measured open circuit S_11_ of the L-matched HSMS-286C diodes at 13.6 MHz for different input power levels from a 50 Ω source (**left**), measured open circuit input impedance at −10 dBm and −40 dBm of the L-matched HSMS-286C diode at 13.6 MHz (**right**).

The measured S_11_ and input impedance at open circuit are shown in Figure 15. There is high return loss and resonance around 13.6 MHz. The circuit input impedance at open circuit conditions is 58 Ω at resonance for both −40 dBm and −10 dBm.

**Figure 16.**
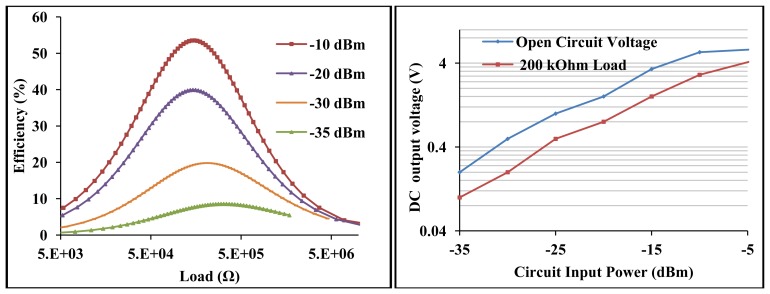
Measured circuit efficiency *versus* load at various input power levels at 13.6 MHz (**left**), measured open circuit voltage and at 200 kΩ load *versus* input power at 13.6 MHz (**right**).

The efficiency and voltage sensitivity of the high voltage sensitive wireless EM harvester are shown in Figure 16.

The maximum measured efficiency at −30 dBm is 20% for ∼200 kΩ load and an open circuit voltage of 0.5 V. At −10 dBm, the maximum efficiency and open circuit voltage are 54% and 5.4 V respectively. At the optimal load of ∼200 kΩ, the detected voltage is 0.2 V and 2.9 V at −30 dBm and −10 dBm respectively. The open circuit voltage gain is 100 at −30 dBm and 108 at −10 dBm.

Even though the RF to DC converter presented in Section 2.5.3 is the same as the L-match circuit realized with the HMSM-286C diodes at 13.6 MHz, the voltage gain is increased by a factor of 4 due to the large difference between the diodes (and load) resistance and source resistance so that at matched conditions high voltage gain occurs. The loaded *Q* of the L-matched circuit is 30 which results in small frequency bandwidth just like a PI-matched diode rectifier presented in our earlier work [40]. From this result and the results from our earlier presented PI-matched EM harvester, it can be inferred that all high loaded *Q* RF to DC circuits have high selectivity but not all highly selective RF to DC circuits have high loaded *Q*. The voltage sensitivity of the matched HSMS-286C diode at 13.6 MHz can be improved if its resistive impedance is not lowered by the flicker noise.

## 3. Wireless EM Power Harvester

**Figure 17.**
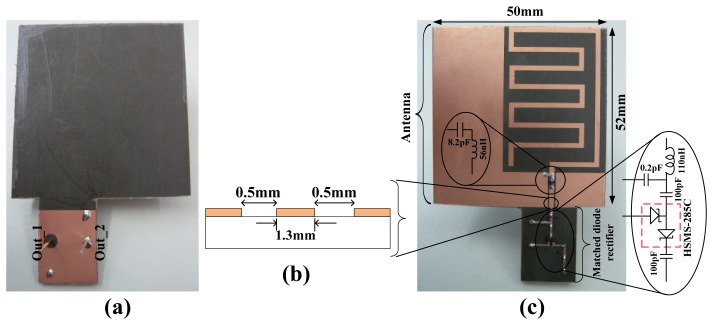
Rectenna realized on a Duroid 5880, 1.57 mm substrate. (**a**) Backside of the rectenna, (**b**) cross-section of antenna output coplanar stripline dimensions (**c**) frontside of the rectenna.

A wireless EM harvester, consisting of a rectifying antenna (*rectenna*) was designed to find a compromise between size and performance of its antenna. The rectenna is shown in Figure 17.

The antenna (planar) part of the rectenna is based on our earlier work [41]. In contrast to the earlier presented antenna, this rectenna is realized on a Duroid [42] substrate of thickness 1.57 mm. Duroid 5880 has lower loss tangent of 0.0004 at 1 MHz compared to 0.025 at 1 MHz for FR4. This means there is less loss in the transmission of signals on a Duroid PCB at this frequency range. The antenna part is fabricated to resonate around 434 MHz; hence its dimensions of 5 × 5.2 cm make it electrically small. The antenna is tuned with a chip inductor and a capacitor to achieve the resonance frequency around 434 MHz [Figure 17(c)]. This is done at a cost of reduced antenna radiation efficiency. An antenna is one of the few components the size of which is related to the operating frequency. Thus, if the size of an antenna is fixed, resonance frequency reduction of the antenna can only be achieved with penalty factors [10]. The antenna's output impedance is tuned with the dimensions of the coplanar stripline as shown in Figure 17(b).

**Figure 18.**
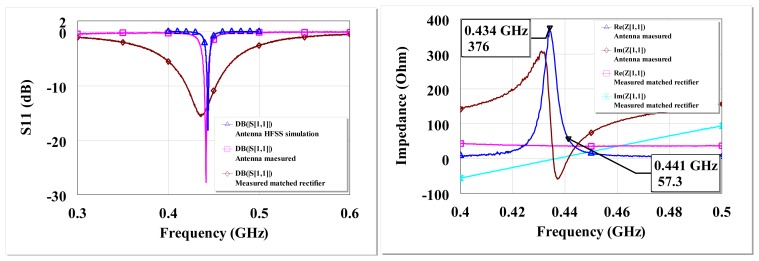
Antenna HFSS simulated, antenna measured, and measured L-matched diode rectifier S_11_ on a Duroid 5880 PCB for −30 dBm input (**left**), Measured open circuit input impedance of antenna and rectifier at −30 dBm input (**right**).

HFSS [43] was used to simulate the presented antenna and to find the correct capacitive and inductive components for frequency tuning before the optimized design was fabricated. The simulated antenna resonances occur at 438 MHz and 445 MHz. At these frequencies, the radiation efficiency is 20% and a peak gain of −6 dBi. The rectifying part of the rectenna consists of L-matched HSMS-285C diodes (Figure 17(c)). The L-matched HSMS-285C part of the rectenna can be engineered to be as small as possible if required. The separate parts of the rectenna were characterized by terminating their ends and measuring the individual reflection coefficients just like the power converters presented in Section 2. Figure 18 shows the measured antenna and matched rectifier individual S_11_ and impedance. Figure 18 (left) also show the HFSS simulated S_11_ results. From Figure 18 (right), the measured antenna resonance where the input impedance is at maximum is ∼434 MHz. At ∼434 MHz, the antenna input resistance is 376 Ω and the reactive impedances approach their extreme (*so called anti-resonance*). The other resonance occurs when the input resistance is ‘finite’ and the reactive impedance is zero; at ∼441 MHz. The input resistance is 57 Ω at ∼441 MHz. The rectifier circuit is matched for the antenna's resistance at ∼441 MHz.

### 3.1. EM Range Results and Discussion

**Figure 19.**
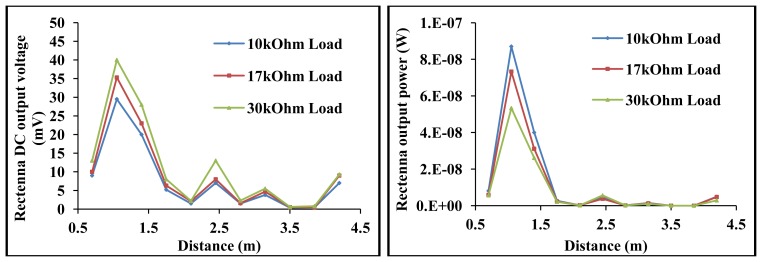
Rectenna receiving range performance by sending 17 dBm (50 mW) at a gain of −6 dBi at 437 MHz. Output DC voltage *versus* receiving distance for different loads (**left**), loads output power *versus* receiving distance (**right**).

At far field between wireless EM transmitting and receiving antenna, the coupling mechanism between the transmitting and receiving antenna is neither capacitive nor inductive as is the case for the RF to DC converters. The coupling is radiative which can be described by the Friis equation of transmission on the assumption that the transmitting and receiving antenna are in free space [44]. A modified Friis equation for a transmitting and receiving antenna at far-field (R ≫ λ and R ≫ transmitting antenna largest dimension) to each other at a specified direction is given by Equation (43) [45]. Equation (43) assumes real world open space conditions:

(43) $$\frac{P_{r}}{P_{t}}=F_{\text{envt}}G_{t}G_{r}{\left(\frac{\lambda }{4\pi R}\right)}^{2},$$

where *Pr* is the power at the receiving antenna port, *Pt* is the power supplied at the transmitting antenna port, *F_envt_* is a factor accounting for environmental effects as such ground reflections among others, *Gt and Gr* are the transmitting and receiving antenna gain (*at specified direction*) respectively. *R* is the distance between the transmitting and receiving antenna and *λ* is the wavelength of the transmitting EM wave. The rectenna receiving range measurements were carried out in an open space (*hall*) with the antennas 2 m above ground level. The transmitting and receiving antennas were arranged in the direction of their peak gain. The rectenna range performance is shown in Figure 19. According to Equation (43), the efficiency of RF power transferred between a sending and receiving antenna depends on controllable factors like the gain of the antennas in the arranged direction and the radiation efficiency of the antennas. Since the receiving/transmitting antenna's incorporated in remote harvesters for sensor powering are normally small in relation to their operating frequencies, they tend to be less efficient.

The efficiency of the rectenna's antenna is ∼20% at resonance. A ‘perfectly’ matched RF to DC power converter operating in its square law region has efficiencies in the region of 20% as depicted in Section 2. The transmitting antenna was the same as the antenna incorporated in the rectenna. By transmitting the EM power with a small antenna (5 cm × 5.2 cm) at 437 MHz with efficiency of ∼20% and at a gain of −6 dBi, the power delivered by the rectenna is generally low at far-field from the transmitter as can be seen in Figure 19. A mediocre transmitting antenna was used to transmit the EM waves due to limitations in the European Union about transmitting EM power at certain frequencies; so the goal in the rectenna range experiment is to show the lowest limit functionality of such a harvester. At 4.2 m from the electrically small transmitting antenna transmitting at 17 dBm, the rectenna harvested DC voltage and power are 9 mV and 5 nW respectively for 10 kΩ load. It can be seen from Figure 19 that the harvested voltage/power generally degrades as an inverse square of distance from transmitter as described by Friis equation. The measured received power however alternate along this *R*^−2^ fit as shown in Figure 19. This anomaly is accounted for by *F_envt_* [Equation (43)] as influence of ground reflections and polarization in real world open field measurements [45]. For any particular distance *R*, the signals reflected from ground can be constructive with the direct signal to the rectenna, in which case the measured power may be higher than that predicted by the original Friss equation as in [44]. The ground effect can also be destructive, in which case the measured power will be lower than what is predicted by the original Friis equation.

## 4. Conclusions

Optimization of Schottky diode-based RF to DC power converters using different matching techniques for wireless EM energy harvesting applications is presented. Using scattering parameters for small signal modeling, it is shown that wireless EM harvesters can be generally described as coupled resonators with efficiencies and maximum voltage sensitivity depending mostly on the source and load resistances under matched conditions. The analytical models allow systematic control in the design of passive wireless EM harvesters. Based on these analyses, a rectenna is built and tested for lower limit functionality from harvesting ambient EM waves. The analysis presented in this work may also be applied to optimize derivatives of wireless EM harvesters like RFID tags, NFC, wireless chargers *etc.*, for efficient powering of their sensors or integrated circuits. Generally, most energy harvesters and their matched loads can be described as coupled resonators and thus may be optimized with the methods presented in this work.

## Figures and Tables

**Figure A1.**
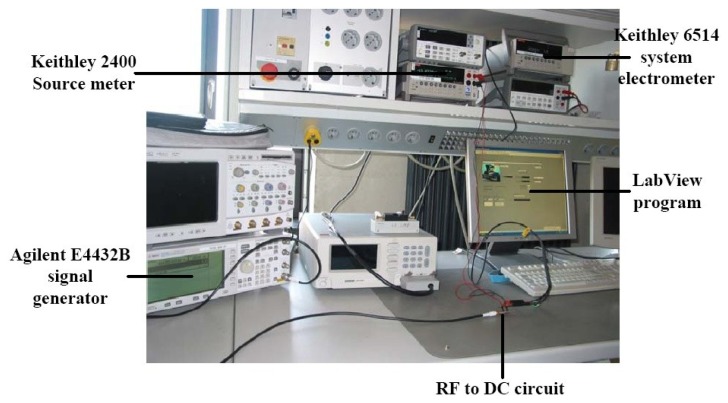
RF to DC Power converter characterization setup.

**Figure A2.**
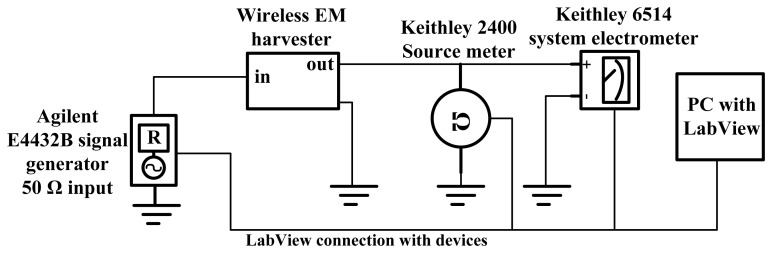
RF to DC power converter characterization circuit.

## Appendix A: Measuring Setup for RF Rectifier Efficiency and Voltage Sensitivity

The measuring setup is as shown in Figure A1.

The RF to DC circuit efficiency and voltage sensitivity measurements were made with a Keithley 2400 source meter and Keithley 6514 system electrometer with an Agilent E4432B signal generator providing 50 Ω RF signal into the circuit board.

The closed circuit current drawn by the RF to DC power converter (*without load*) from the generator is first determined by the Keithley 2400 source meter; then starting from this current, the value of the current is decreased at set intervals to creates virtual load resistances to the circuit for up to a lowest current of 0.1 μA. The 6514 system electrometer is used to measure the output voltage. The number of data point is set through LabView [46] as well as the measurements. Additionally open circuit voltage or at specific loads and frequency sweep can be made through the LabView program. At −40 dBm input power and below, the detected voltages and currents were difficult to measure accurately with the measuring setup; hence measurements were made up to a minimum of −35 dBm input power. The circuit layout for the efficiency and voltage sensitivity measurements is schematically shown in Figure A2.

## References

[1] Fischer J. (2009). NFC in cell phones: The new paradigm for an interactive world [Near-Field Communications]. IEEE Commun. Mag..

[2] Shameli A., Safarian A., Rofougaran A., Rofougaran M., de Flaviis F. (2007). Power harvester design for passive UHF RFID tag using a voltage boosting technique. IEEE Trans. Microw. Theor. Tech..

[3] Karthaus U., Fischer M. (2003). Fully integrated passive UHF RFID transponder IC with 16.7-μW minimum RF input power. IEEE J. Solid-State Circuits.

[4] Lee S.H., Jin I.S. (2011). Interoperation of an UHF RFID reader and a TCP/IP device via wired and wireless links. Sensors.

[5] Mandal S., Sarpeshkar R. (2007). Low-power CMOS rectifier design for RFID applications. IEEE Trans. Circuits Syst. I: Regul. Papers.

[6] Yu H., Bashirullah R. A Low Power ASK Clock and Data Recovery Circuit for Wireless Implantable Electronics.

[7] Hannan M.A., Abbas S.M., Samad S.A., Hussain A. (2011). Modulation techniques for biomedical implanted devices and their challenges. Sensors.

[8] Cho N., Song S.-J., Lee J.Y., Kim S., Kim S., Yoo H.-J. A 8-μW, 0.3-mm2 RF-powered Transponder with Temperature Sensor for Wireless Environmental Monitoring.

[9] Brown W.C. (1984). The history of power transmission by radio waves. IEEE Trans. Microw. Theor. Tech..

[10] Mickle M.H., Mi M., Mats L., Capelli C., Swift H. (2006). Powering autonomous cubic-millimeter devices. IEEE Antennas Propag. Mag..

[11] McSpadden J.O., Chang K. A Dual Polarized Circular Patch Rectifying Antenna at 2.45 GHz for Microwave Power Conversion and Detection.

[12] Sample A., Smith J.R. Experimental Results with Two Wireless Power Transfer Systems.

[13] Umeda T., Yoshida H., Sekine S., Fujita Y., Suzuki T., Otaka S. (2006). A 950-MHz rectifier circuit for sensor network tags with 10-m distance. IEEE J. Solid-State Circuits.

[14] Le T., Mayaram K., Fiez T. (2008). Efficient far-field radio frequency energy harvesting for passively powered sensor networks. IEEE J. Solid-State Circuits.

[15] Yuan F. (2011). CMOS Circuits for Passive Wireless Microsystems.

[16] Zbitou J., Latrach M., Toutain S. (2006). Hybrid rectenna and monolithic integrated zero-bias microwave rectifier. IEEE Trans. Microw. Theor. Tech..

[17] Ungan T., Le Polozec X., Walker W., Reindl L. RF Energy Harvesting Design Using High Q Resonators.

[18] Ungan T., Freunek M., Muller M., Walker W.D., Reindl L.M. Wireless Energy Transmission Using Electrically Small Antennas.

[19] Boquete L., Rodríguez-Ascariz J.M., Barea R., Cantos J., Miguel-Jiménez J.M., Ortega S. (2010). Data acquisition, analysis and transmission platform for a pay-as-you-drive system. Sensors.

[20] Heikkinen J., Salonen P., Kivikoski M. Planar Rectennas for 2.45 GHz Wireless Power Transfer.

[21] Akkermans J.A.G., van Beurden M.C., Doodeman G.J.N., Visser H.J. (2005). Analytical models for low-power rectenna design. IEEE Antenn. Wireless Propag. Lett..

[22] Hagerty J.A., Helmbrecht F.B., McCalpin W.H., Zane R., Popovic Z.B. (2004). Recycling ambient microwave energy with broad-band rectenna arrays. IEEE Trans. Microw. Theor. Tech..

[23] Harb A. (2011). Energy harvesting: State-of-the-art. Renew. Energ..

[24] Vullers R.J.M., van Schaijk R., Doms I., van Hoof C., Mertens R. (2009). Micropower energy harvesting. Solid-State Electron..

[25] Sah C.-T. (1991). Fundamentals of Solid-State Electronics.

[26] Wetenkamp S. Comparison of Single Diode *vs.* Dual Diode Detectors for Microwave Power Detection.

[27] Cardoso A.J., Schneider M.C., Montoro C.G. Design of Very Low Voltage CMOS Rectifier Circuits.

[28] Cardoso A.J., de Carli L.G., Galup-Montoro C., Schneider M.C. (2012). Analysis of the Rectifier Circuit Valid Down to Its Low-Voltage Limit. IEEE Trans. Circuits Syst. I: Regul. Papers.

[29] Data sheet HSMS-285x.

[30] Data sheet HSMS-286x.

[31] Hsu T.S. (1970). Low-frequency excess noise in metal—Silicon Schottky barrier diodes. IEEE Trans. Electron Devices.

[32] Hastas N.A., Dimitriadis C.A., Dozsa L., Gombia E., Amighetti S., Frigeri P. (2003). Low frequency noise of GaAs Schottky diodes with embedded InAs quantum layer and self-assembled quantum dots. J. Appl. Phys..

[33] Gomila G., Reggiani L., Rubí J.M. (2000). Shot-noise suppression in Schottky barrier diodes. J. Appl. Phys..

[34] Kleinpenning T.G.M. (1979). Low-frequency noise in Schottky barrier diodes. Solid-State Electron..

[35] Lee T.H. (2004). The Design of CMOS Radio-Frequency Integrated Circuits.

[36] Advanced Design System.

[37] Bowick C., Blyler J., Ajluni C.J. (2008). RF Circuit Design.

[38] Joe J., Chia M., Marath A., Ang C. Zero Bias Schottky Diode Model for Low Power, Moderate Current Rectenna.

[39] Visser H.J., Vullers R.J.M., Veld B.O.h., Pop V. Remote RF Battery Charging.

[40] Nimo A., Grgic D., Reindl L.M. (2012). Impedance optimization of wireless electromagnetic energy harvester for maximum output efficiency at μW input power. Proc. SPIE.

[41] Nimo A., Grgic D., Reindl L.M. Electrically small Planner Antenna for Compact Electromagnetic (EM) Wireless Energy Harvester.

[42] (2011). RT-duroid-5870–5880-Data-Sheet.

[43] (2005). HFSS.

[44] Friis H.T. (1946). A Note on a Simple Transmission Formula. Proc. IRE.

[45] Kvaksrud T.I. (2008). Range Measurements in an Open Field Environment, Design Note DN018.

[46] (2009). LabView.

